# Has Tourism Industry Agglomeration Improved the Total Factor Productivity of Chinese Urban Agglomerations?—The Moderating Effect of Public Epidemic

**DOI:** 10.3389/fpubh.2022.854681

**Published:** 2022-03-17

**Authors:** Jianhua Wang, Junwei Ma

**Affiliations:** Business School, Changshu Institute of Technology, Suzhou, China

**Keywords:** tourism industry agglomeration, total factor productivity, public epidemic, moderating effect, urban agglomeration

## Abstract

Industry agglomeration has become a prominent feature of tourism industry development in developed and developing countries and regions in the world. According to the literature analysis, the development of industrial agglomeration has both agglomeration effect and congestion effect. This paper constructs a theoretical and empirical analysis framework for the impact of tourism industry agglomeration on the total factor productivity of Chinese urban agglomerations, and analyze the moderating effect of the public epidemic on this impact. From results of empirical analysis, a U-shaped relationship exists between tourism industry agglomeration and the total factor productivity of Chinese urban agglomerations. The public epidemic positively moderated (enhanced) the negative effect (congestion effect) of tourism industry agglomeration on total factor productivity, and negatively moderated (weakened) the positive effect (agglomeration effect) of tourism industry agglomeration on total factor productivity.

## Introduction

Industry agglomeration development is a worldwide economic phenomenon. Industry agglomeration combines the isolated small and medium-sized enterprises with an innovative development mode to maximize the integration of natural advantages and endowments, and enhance the overall production efficiency and competitive advantage of the industry. There are many industries involved in tourism, and there are many horizontal associations between industries, which is one of the industries with the most obvious agglomeration effect and the most suitable for agglomeration development (1). At the same time, Porter cites clusters examples related to tourism industry as actual clusters. For example, the California wine cluster. Tourism industry agglomeration as a strategic means of regional tourism development can effectively guide the development of regional tourism (2). The South African government used the concept of clusters to guide the country's travel tourism development. Sri Lanka, Ghana, Guyana, Croatia and other countries also tried to establish a tourism industry cluster. In recent years, regional tourism industry agglomeration have emerged continuously, and the industries within the cluster have been concentrated, which has also produced good synergies and innovation effects. Agglomeration has become a prominent feature of the development of tourism industry in developed and developing countries and regions of the world (3).

Since the 1960s, the growth rate of the global tourism economy has generally been higher than that of the global economy. Tourism has gradually developed into the world's largest emerging industry, and has even surpassed the petroleum and automobile industries to become the world's largest industry. Tourist destinations represented by emerging countries continue to appear, and the world's regional center of gravity is shifting to the east. China is the representative of this trend. After 40 years of reform and opening up, China's tourism industry has become an important part of national economy and a basic guarantee for China's sustained economic growth and stable operation (4, 5). According to data from the China Tourism Academy, in 2019, China's tourism industry contributed 11.05% to GDP and 10.31% to national employment. With the rapid development of transportation and information technology, the time and distance between regions are shortened, and the tourism economy between regions is more closely connected. This close connection and interaction has significantly strengthened the mobility of related tourism elements between regions and promoted tourism. The spatial configuration and integration of elements are constantly evolving, thereby accelerating the formation and development of tourism industry agglomeration.

Theory and practice have proved that industry agglomeration will not only produce agglomeration effect that contribute to economic growth, but also generate congestion effect that hinder economic development. There are also differences in the strength of the two effects at different stages of agglomeration changes. Therefore, the strength of the agglomeration effect and the congestion effect of the tourism industry agglomeration (TIA) determine whether it can improve the regional total factor productivity (TFP). In addition, the Williamson hypothesis believes that the agglomeration effect will significantly improve economic efficiency before the economy develops to a critical level, but then, the agglomeration effect turns into a congestion effect, which has a negative effect on economic efficiency (6). From a theoretical point of view, TIA also has agglomeration effect and congestion effect. Under the background of supply-side structural reform in China, the tourism industry, as an important industry to ensure the long-term stability of China's economy, is urgently needed to clarify the mechanism of the relationship between TIA and TFP both in terms of theoretical research and industrial practice. Whether TIA exerts the agglomeration effect or the congestion effect is related to the sustainable development of the tourism industry.

Public health emergencies not only directly cause a certain degree of health damage to the public physically and psychologically, but also have an immeasurable impact on regional development economically and politically. As a public health emergency, the global coronavirus disease (COVID-19) not only poses a serious threat to human life and health, but also has a significant impact on the development of the world's tourism industry. As the epidemic is gradually brought under control, the economy has gradually recovered, but the recovery of the tourism industry has been slower than that of other industries. The World Tourism Council estimates that it will take up to 19 months for the tourism industry to recover. Although the global epidemic has caused the global tourism industry to almost stagnate, the China Tourism Research Institute predicts that China's tourism economy will recover in a “U-shape” in 2020. At present, China's economic and social development is at a critical stage of innovation-driven transformation. In this important evolution process, China's tourism industry has undergone a transformation of “tourist spots–tourist attractions–tourist resorts–national tourist economic zones–global tourism” around core tourism resources. As one of the fastest-growing industries in China, the tourism industry is characterized by its comprehensiveness and strong relevance, which determines its spatial agglomeration characteristics. Therefore, this paper discusses the moderating effect of public epidemic in the relationship between tourism industry agglomeration and total factor productivity of urban agglomerations.

To date, tourism industry research is mainly concentrated in cities, and tourism research for urban agglomerations is also less. This paper constructs a theoretical and empirical analysis framework, taking China's 10 urban agglomerations as examples to test the relationship between TIA and TFP, and analyze the moderating effect of the public epidemic. Different from previous studies, this paper makes possible contributions in four aspects: First, this paper theoretically analyzes the mechanism of TIA on TFP. Previous studies have focused on the impact of manufacturing industry agglomeration on regional economic efficiency, but there are few studies with respect to the impact of TIA on TFP. Second, based on panel data model and econometric methods, the non-linear relationship between TIA and TFP of urban agglomerations is empirically tested. Third, this paper discusses the moderating effect of public epidemic in the relationship between TIA and TFP from the perspective of exogenous shocks. Fourth, the sample selected in this paper is multiple urban agglomerations rather than cities or a single urban agglomeration. Because the tourism industry has a high degree of regional openness and industrial relevance, urban agglomeration has become an important space carrier for the development of regional tourism industry. The urban agglomeration has a high degree of agglomeration characteristics, and the agglomeration characteristics of the tourism industry in urban agglomerations are more obvious.

The next section of this five-section paper reviews the literature and develops research hypotheses. The third section describes materials and empirical methods. The four section interprets the results. The final section summaries the major findings, contributions, followed by suggestions for future work.

## Literature Review

With the rapid development of urban agglomerations, more and more scholars pay attention to the phenomenon of urban agglomerations, and carry out theoretical and empirical research on the economic development of urban agglomerations. It is believed that urban agglomerations are increasingly becoming one of the most important modes of economic competition. The economic efficiency of urban agglomerations is obviously better than that of non-urban agglomerations (7). As urban agglomerations have better transport and communication infrastructure, multinational corporations are connected to each other, knowledge flows and relationships are easier to establish, and companies can access a diverse and specialized workforce and global business services, which improve the economic efficiency and competitiveness of urban agglomerations (8–10). In addition, closer ties between cities within the urban agglomeration can better address their economic development issues, resulting in better economic development of the urban agglomeration (11). Thus, urban agglomerations can achieve better “centralized dispersion” (12).

Industry agglomeration development is a worldwide economic phenomenon. Since the American economist Marshall proposed the theory of industry agglomeration in the late twentieth century, industry agglomeration and economic development have been the focus of research. A series of new economic geography theoretical models reveal that industry agglomeration and economic growth are essentially mutually reinforcing endogenous processes. Industry agglomeration is beneficial to overall economic growth, and geographical location will affect economic growth (13–17). However, no consensus conclusions have been reached on relevant empirical research. Some scholars have confirmed that agglomeration does have a significant effect on industrial growth or regional economic efficiency (18–21). However, some scholars have found that the role of agglomeration in promoting regional economic growth is limited (22), and even hinders industrial growth or regional economic growth (23, 24). Some scholars believe that there may not be a simple linear correlation between agglomeration and growth. The agglomeration effect and congestion effect of industry agglomeration have been proved in related research (25–27).

The International Cluster Consortium pointed out TIA was a geographical concentration of tourism enterprises and related organizations which cooperate for a common goal and establish close ties. This enables the region to gain overall competitive advantage (28). This concept inherits Porter's idea of taking regional scale as a unit to obtain regional competitiveness as the fundamental goal. Scholars mostly agree with Porter's view when conducting research on TIA (29). Scholars believe that the combined effects of internal and external causes promote TIA. When scholars discuss the external driving factors of TIA, they mainly involve resource endowment, market demand, location conditions, and government promotion (30). Compared with the external driving mechanism, the internal driving mechanism is more concerned by researchers, including the economies of scale and scope, flexible specialization, innovative learning, externality and so on (31–33).

Whether it is TIA as a result of regional development or as a strategy for regional economic development, TIA will have a significant impact on TFP. Regarding the relationship between TIA and TFP, relevant research has been carried out. Most scholars believe that TIA has a positive effect on tourism economic development or economic efficiency (29, 34–37). For example, TIA can reduce the economic leakage of tourist destinations (38), prevent the decline of local tourism (39), and ultimately enhance the regional tourism competitiveness (32). Li and Liu (40) found that TIA could significantly improve the overall efficiency, pure technical efficiency, and scale efficiency of Chinese provincial tourism industry. However, some scholars believe that China's tourism industry at the current stage has a low level of agglomeration, and tourism industry agglomeration has no positive effect on the economic development (41). Some scholars believe that the prosperity and development of tourism industry is an industrialization process, because the income brought by tourism has attracted more manufacturers to join. On the other hand, the development of tourism industry is a process of de-industrialization because tourism industry attracts the labor force of the manufacturing industry (42). Based on theoretical analysis, this paper believes that China's TIA has both agglomeration and congestion effect. Therefore, this paper proposes the following hypothesis:

**H1: It is a Non-Linear Relationship Between TIA and TFP of Urban Agglomerations**.

Scholars have studied the impact of public emergency shocks on the micro and macro levels. Kaplanski and Levy (43) found that the loss of aviation stock market value caused by air crash events was much greater than the real economic loss of aviation companies caused by air crashes. Zhao (44) discussed the correlation between the information disclosure of pharmaceutical companies and the individual stock market response in emergencies based on the SARS outbreak. The study found that the positive driving effect of pharmaceutical epidemic disclosure on individual stock market effects was affected by the “conspicuous” effect of information disclosure degree of influence. The research on the COVID-19 epidemic mainly focuses on the macro level, and discusses the impact of the COVID-19 epidemic on the macroeconomic operation and its transmission path (45, 46), and the choice of government intervention strategies under the impact of the COVID-19 epidemic (47, 48). The empirical analysis results of Shen et al. (49) shown that the COVID-19 epidemic had a significant negative impact on the performance of Chinese listed companies, mainly in the reduction of investment scale and operating profit. Luo (50) found that compared with state-owned enterprises and large enterprises, the COVID-19 epidemic had a greater impact on private enterprises and small and micro enterprises, and business risks may also be transmitted along the supply chain and guarantee chain, causing localized crises. Huang et al. (51) found that the epidemic had significantly reduced the willingness of enterprises to carry out economic activities in the future. Tang et al. (52) used the SARS epidemic in 2003, the international financial crisis in 2008, and the earthquake in Japan in 2011 to investigate the impact of exogenous shocks on corporate investment and value chains, and found that supply-side and demand-side shocks affected corporate investment and value. It was found that there were differences in the impact of the chain, and the government's response policies would resist the negative impact of exogenous shocks on corporate investment to a certain extent. Xie et al. (53) found that the impact of the COVID-19 pandemic had a significant positive impact on corporate innovation bias, while credit financing support negatively moderated the positive relationship between the COVID-19 pandemic and corporate innovation bias. Through literature review, it is found that public emergencies not only have short-term impacts, but also have medium-term and long-term impacts on regional economic development. Different from one-time, localized shocks such as terrorist attacks and natural disasters, the characteristics of the COVID-19 epidemic, such as its strong transmission, wide epidemic range and long duration, make the impact of this epidemic lasting and far-reaching. Therefore, this paper proposes the following hypothesis:

**H2: Public epidemic plays a moderating role in the relationship between TIA and TFP of urban agglomerations**.

At the same time, some other factors of tourism industry will also have an impact on TFP. Studies have pointed out that tourism resources are an important factor in the development of natural environment-dependent tourism (54). The elements or resources of tourism industry development include many types, such as demand factors, natural resources, technical resources, and service resources. The influence of these factors on tourism economy or regional economic development has also attracted the attention of scholars. Based on the research of scholars, this paper believes that residents' disposable income (DI) (55), technological innovation (TP) (56), marketization institution (MI) (57), transportation infrastructure (TI) (58) will also affect TFP. Therefore, *this paper selects these factors* as control variables.

## Materials and Methods

### Study Area

China's “13th Five-Year Plan” issued by the National Development and Reform Commission of China proposed to promote the sustainable development of some key urban agglomerations. Taking into account the differences in the development level of regional economy and urban agglomerations, *this paper selects 10 urban agglomerations* as study samples. Typical urban agglomerations are as follows: Beijing-Tianjin-Hebei, Yangtze River Delta, Pearl River Delta, Shandong Peninsula, West Taiwan Strait, Mid-southern Liaoning, Central Plains, Middle Yangtze River, Chengdu-Chongqing, and Guanzhong urban agglomerations, which include a total of 122 cities. These urban agglomerations are the most fundamental areas supporting China's land development and also play a vital role in China's participation in global competition. During the period of 2004–2019, the tourism revenue of these 10 urban agglomerations in China accounted for about 10% of the regional GDP, and the highest proportion reached 20%. Geographically, these 10 urban agglomerations involve national and regional study samples, which also involve eastern, middle, and western economic district in China with gradient differences, and can better represent the economic development level and characteristics of the three regions in China.

### Data Sources

The data statistics of the sample are from 2002 to 2021. The sample includes public health emergencies such as SARS that began in 2003 and the COVID-19 epidemic that began in 2020. In this paper, most statistical data were derived from the authoritative statistical yearbooks, including the 2003–2021 China Urban Statistical Yearbook, the 2003–2021 China Statistical Yearbook on Science and Technology, and the 2003–2021 China Statistical Yearbook. Tourism revenue data for each region comes from the annual statistical bulletin of national economic and social development in each province or city in China (2003–2021). The data on the public epidemic comes from the World Pandemic Uncertainty Index (https://worlduncertaintyindex.com/data/). The marketization institutiou indicator of urban agglomerations were derived from the China Marketization Index Report published by Wang et al. (59). The data for all variables in 2021 are forecast values.

### Methods

Based on literature research, this paper argues that the impact of TIA on TFP of urban agglomeration is also divided into agglomeration effect and congestion effect. The agglomeration and congestion effects of TIA will be accompanied by the entire period of TFP changes in urban agglomerations. Therefore, in the model of TIA and TFP, this paper expresses the change process by means of the square term of TIA ${\left(TIA_{it}\right)}^{2}$. If the coefficient before the square term of TIA is positive, then TIA in this period will promote TFP of urban agglomerations. If the coefficient is negative, it indicates that TIA in this period has hindered TFP of urban agglomerations. In order to analyze in detail the current stage of China's TIA on TFP of urban agglomerations, this paper constructs the panel data model as follows.


(1) $$\begin{array}{r} TFP_{it}=\beta_{0}+\beta_{1}\;TIA_{it}+\beta_{2\;}{\left(TIA_{it}\right)}^{2}+\gamma \;X_{it}+\mu_{i}+\lambda_{t}+\varepsilon_{it} \end{array}$$

where *TFP*_*it*_ is TFP of urban agglomeration *i* in the year *t*, and *TIA*_*it*_ is the level of TIA of urban agglomeration *i* in the year *t*. *X*_*it*_ represents the control variables, for example, residents' disposable income (DI), technological innovation (TP), marketization institution (MI), transportation infrastructure (TI), etc. μ_*i*_ is the individual effect, λ_*t*_ is the time effect, and ε_*it*_ is the random error term. *i* =1, 2,..., 10, representing 10 urban agglomerations; *t* = 1, 2,..., 20, representing time 2002, 2003,..., 2021.

In order to test the moderating effect of the public epidemic, this paper adds a new variable Discussion about Pandemics Index (DPI) to the new model. The addition of this explanatory variable can help to analyze the moderating role of DPI in the relationship between TIA and TFP.Therefore, this paper adds the intersection of TIA and DPI in the above model (1) to further test the impact of TIA on TFP. The panel data model was modified as follows.


(2) $$\begin{array}{r} TFP_{it}=\beta_{0}+\beta_{1}\;TIA_{it}+\beta_{2}\;{\left(TIA_{it}\right)}^{2}+\beta_{3}\;\left({TIA}_{it}*DPI_{t}\right)\\ \;+\beta_{4}\;{\left(TIA_{it}\right)}^{2}*DPI_{t}+\gamma {\;X}_{it}+\mu_{i}+\lambda_{t}+\varepsilon_{it} \end{array}$$

Calibrated variables in the panel data models are shown in Table 1.

**Table 1:** Calibrated variables.

**Variables**	**Abbr**.	**Description**
Total factor productivity	TFP	Total factor productivity of urban agglomerations
Tourism industry agglomeration	TIA	The ratio of tourism industry income to gross domestic product in urban agglomerations to the national level
Discussion about pandemics index	DPI	The discussion about pandemics index at the country level
Residents' disposable income	DI	Residents' disposable income
Technological innovation	TP	The ratio of the total number of patent application to the total land area of urban agglomerations
Marketization institution	MI	The marketization index of urban agglomerations
Transportation infrastructure	TI	The ratio of the total length of the road, railway and inland waterway to the total land area of urban agglomerations

### Calibrated Variables

The calibrated variables involved in the regression equation mainly include TFP, TIA, DPI, DI, TP, MI and TI.

#### Dependent Variable: TFP

TFP measurement methods include growth kernel algorithm, frontier analysis and index method. As a frontier analysis method, Data Envelopment Analysis (DEA) uses the optimization method to determine the weight of various input factors endogenously, avoiding the specific expression of the relationship between input and output, and eliminating the interference of many subjective factors on the measurement method. It also has advantages such as no relationship with market price, and it is especially suitable for TFP evaluation of complex economies.

This paper uses the DEA-Mamquist model to measure TFP of urban agglomerations. According to the classical Cobb-Douglas production function:


(3) $$\begin{array}{r} Q=AL^{\alpha }K^{\beta } \end{array}$$

where the two most important input factors in economic growth are labor *L* and capital *K*. In macroeconomic output, natural resources are also a key production factor, the most important of which is the land element. The output of efficiency is the macroeconomic output of urban agglomerations. Therefore, TFP measurement model of urban agglomerations includes three input indicators and one output indicator. The first input indicator is labor factor, measured by the total number of employed people in urban agglomerations. The second input indicator is the capital factor, measured by the total capital stock of urban agglomerations in the current year. The third input indicator is the natural factor, which mainly refers to the input of the land elements of urban agglomerations, measured by the total land area of urban agglomerations. The capital stock is estimated using Goldsmith's Perpetual Inventory Method (PIM). The basic estimation formula is as follows.


(4) $$\begin{array}{r} K_{t}=\left(1-\delta_{t}\right)K_{t-1}+I_{t} \end{array}$$

where *K*_*t*_ and *K*_*t*−1_ represent the regional capital stock in period t and t-1, respectively, δ_*t*_ is the capital depreciation rate in period *t*, and *I*_*t*_ is the investment amount in period t.

The DEA-Malmquist model can be used to measure the change in TFP of China's 10 urban agglomerations from 2002 to 2021. TFP of an urban agglomeration can be expressed as follows.


(5) $$\begin{array}{r} TFP_{it}={TFP_{it-1}\times TFPCH}_{it} \end{array}$$

where *TFP*_*it*_ is TFP of urban agglomeration *i* in the year *t*. *TFPCH*_*it*_ is the Malmquist index of urban agglomeration *i* in the year *t*, which is just an index of change rate, not equal to *TFP*_*it*_. This paper refers to the literature research, and the TFP of the current year is expressed by multiplying the TFP of the previous year and the TFPCH of the current year. *This paper sets 2001 as the base* period, that is, *TFP*_*i*2001_ = 1. *i* = 1, 2,..., 10, representing 10 urban agglomerations; *t* =2002, 2003,..., 2021.

#### Core Independent Variable: TIA

Under normal circumstances, the degree of industrial agglomeration is mainly considered in two aspects: first, the concentration of industry in geographic location, that is, the degree of specialization of industry in a specific region; second, the relationship between related industries in industrial agglomeration, that is, the degree of relevance between industries. At present, the measurement methods of industrial agglomeration mainly include industry concentration, location quotient method, Gini coefficient method, and Hefendale index. Through the comparison of various research methods and the availability of relevant data, *this paper selects the location* quotient index to measure the level of TIA. The location quotient index has been recognized by many scholars in the study of TIA at different regional scales (60–64). The location quotient index can not only fully reflect the spatial distribution of regional industrial factors and the intensity of regional industry development, but also visually reflect the agglomeration level of relevant formats in different regions and the specialization level of different industries. This paper uses the location quotient method to measure the level of China's TIA. The calculation formula is as follows.


(6) $$\begin{array}{r} TIA_{it}=\frac{\frac{ttr_{it}}{gdp_{it}}}{\frac{ttr_{t}}{gdp_{t}}} \end{array}$$

where *TIA*_*it*_ is the location quotient index of the tourism industry of urban agglomeration *i* in the year *t*, which measures the ratio of tourism industry income to gross domestic product (gdp) in urban agglomerations to the national level. If the value of *TIA*_*it*_ is greater than 1, it indicates that TIA in this urban agglomeration is obvious, and the larger the value, the higher the level of agglomeration. *ttr*_*it*_ is the tourism income of urban agglomeration *i* in the year *t*, *gdp*_*it*_ is the gross domestic product (gdp) of urban agglomeration *i* in the year *t*, *ttr*_*t*_ is the national tourism income in the year *t*, *gdp*_*t*_ is the national gdp in the year *t*. *i* = 1, 2,..., 10, representing 10 urban agglomerations; *t* = 2002, 2003,..., 2021.

#### Core Variable: DPI

This paper aims to examine the moderating effect of the public epidemic in the relationship between TIA and TFP. For this purpose, we use the dataset of World Pandemic Uncertainty Index. This dataset includes the World Pandemic Uncertainty Index and an index that measures discussion about pandemics at the global and country level. We choose the Discussion about Pandemics Index (DPI) to measure pandemic uncertainty or public pandemic, and convert monthly data to annual data.

#### Control Variables

Due to the characteristics of the development of tourism industry, resource accumulation factors of tourism industry will also affect TFP of urban agglomerations. Therefore, *this paper selects resource accumulation* factors such as residents' disposable income (DI), technological innovation (TP), marketization institution (MI) and transportation infrastructure (TI) as control variables. Among them, the data of DI comes from the China Statistical Yearbook. TP is measured by the ratio of the total number of patent application to the total land area of urban agglomerations. Referring to the practices of other scholars, this paper uses the marketization index in the “China Marketization Index Report” published by Wang et al. (59) as the maketization institution variable (MI). Therefore, MI index of each urban agglomeration is the arithmetic mean of the corresponding marketization index of the provinces or cities included in the urban agglomeration. TI is measured by the ratio of the total length of the road, railway and inland waterway to the total land area of the urban agglomeration.

## Results

According to the theoretical hypotheses and the panel data model, the regression Equations (1) and (2) are estimated.

### Stationarity Test of Variables

Before the empirical analysis, in order to prevent the phenomenon of pseudo-regression, it is necessary to test the stability of each index. In this paper, the four kinds of stationarity test methods of Levin-Lin-Chu panel unit root test (LLC), Im-Pesaran-Shin panel unit root test (IPS), Fisher-Augmented Dickey-Fuller test (ADF-Fisher) and Fisher-Phillips-Perron test (PP-Fisher) are used to ensure the accuracy of the test conclusion. According to the results of the four test statistic of each variable sequence, it is found that after the first-order difference is involved in the initial variables of the model, all variables pass the 10% significance test. Therefore, the variables in the model are first-order monotonic, that is, obey the *I* (1) process. The panel data is stable, and the co-integration relationship between the dependent variable and the independent variables can be tested before the regression analysis.

### Co-integration Test Between Variables

The Pedroni co-integration test method is the most commonly used test method, which can provide multiple test statistics at the same time, thus enhancing the scientificity of the test conclusion. The co-integration test results of the dependent variable and the independent variables are shown in Table 2.

**Table 2:** Co-integration test results of TFP and independent variables.

**Test T**	***TFP***
Modified Phillips-Perron	3.6655*** (0.0001)
Phillips-Perron	−1.5101* (0.0655)
Augmented Dickey-Fuller	−3.2888** (0.0005)

*, **, ***Indicate that the variable is significant at 10, 5, and 1% confidence level, respectively.

From Table 2, it can be found that the Modified Phillips-Perron, Phillips-Perron, and Augmented Dickey-Fuller statistics of *TFP* all reject the null hypothesis that “there is no co-integration relationship”. Therefore, it can be concluded that there is a co-integration relationship between *TFP* and the independent variables. Therefore, this paper can select panel regression model (1) and (2) to analyze the impact of TIA on TFP of urban agglomerations.

#### Regression Results

According to the research hypotheses and the econometric model, the regression results are shown in Table 3.

**Table 3:** Regression results.

**Index**	***TFP***	
	**(1)**	**(2)**
c	1.5962*** (0.1463)	1.5682*** (0.1517)
*TIA*	−0.1049* (0.0556)	−0.0949* (0.0062)
*TIA^2^*	0.0122** (0.0061)	0.0115* (0.0062)
TIA*DPI		0.0002** (0.0002)
TIA^2*^DPI		−0.00003** (0.00004)
*DI*	−8.29e-08 (6.86e-07)	−1.48e-07 (7.13e-07)
*TP*	0.0456*** (0.0106)	0.0445*** (0.0109)
*MI*	−0.0481*** (0.0144)	−0.0477*** (0.0145)
*TI*	−0.0461*** (0.0143)	−0.0464*** (0.0146)
*F*-Test	5.21 (0.0001)	3.99 (0.0002)
Numbers	200	200
	0.9127	0.9033

*, **, *** indicate that the variable is significant at 10, 5, and 1% confidence level, respectively.

*The values in parentheses are the corresponding standard error*.

*The selection of the model is mainly marked by F-test, and the corresponding statistical value and significance level are marked*.

By analyzing the estimated results of the model, this paper gets some basic results.

First, the impact of TIA on TFP of Chinese urban agglomerations is both agglomeration and congestion effects. From the estimation results of the model (1) and (2), the estimated coefficient of *TIA* is significantly negative, and the estimated coefficient of *TIA*^2^ is significantly positive. This result indicates that a U-shaped relationship exists between TIA and TFP of Chinese urban agglomerations. The hypothesis H1 passes the test, and the result is not consistent to Williamson hypothesis (inverted “U” curve). That is to say, in the early stage, TIA hinders TFP of Chinese urban agglomerations. After a certain stage of development, TIA will promote TFP of Chinese urban agglomerations. By calculation, the turning point is equal to 3.93. This conclusion is not consistent with some scholars. According to some scholars' research, China's TIA on TFP of urban agglomerations has a continuous agglomeration effect. As the resources of the service industry continue to be concentrated in one region, upstream and downstream enterprises in the production chain can cooperate effectively. With the convenience of transportation, enterprises and surrounding organizations vigorously cooperate to improve production efficiency, and thus continue to promote TFP of various regions.

**Figure 1:**
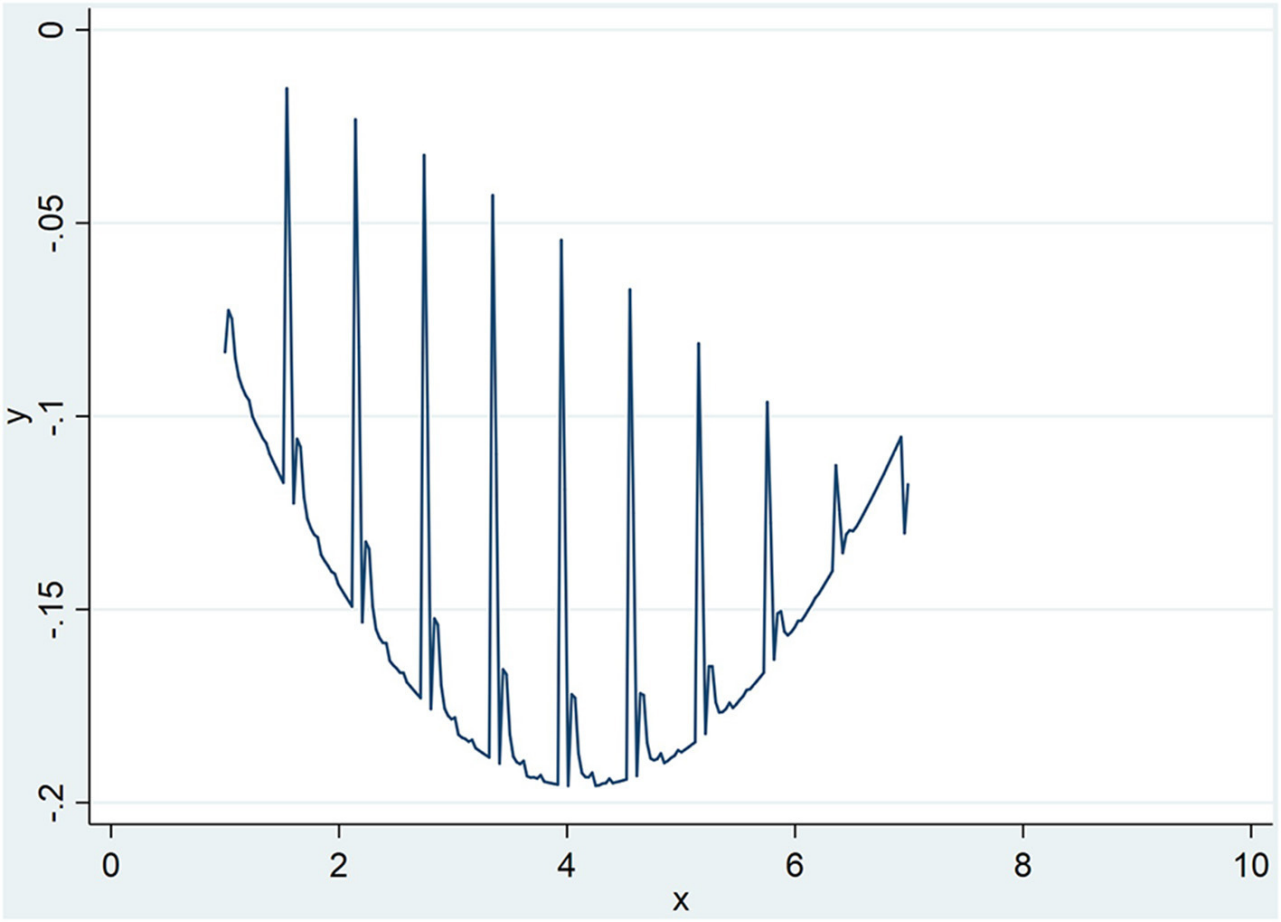
U-shape curve change under different level of DPI.

Second, public epidemic plays a moderating role in the relationship between TIA and TFP of urban agglomerations. From the test results of model (2), the intersection of TIA and DPI (TIA^*^DPI; TIA^2*^DPI) passes the significance test. This shows that the public epidemic significantly moderates the relationship between TIA and TFP of urban agglomerations. The hypothesis H2 passes the test. The public epidemic positively moderated (enhanced) the negative effect of TIA on TFP of urban agglomerations, and negatively moderated (weakened) the positive effect of TIA on TFP of urban agglomerations. And Figure 1 is shown how the U-shape curve change under different level of DPI.

Third, the resource accumulation factors of tourism industry also have an important impact on TFP of urban agglomerations. Among the independent variables, the coefficient of DI of urban agglomerations in the model is negative, but fails the significance test. TP is significant at the 1% statistical level, and the coefficient is positive. This indicates that the level of technological innovation can promote TFP of urban agglomerations. MI is significant at the 1% statistical level, and the coefficient is negative. This indicates that the marketization level is not conducive to TFP of urban agglomerations. This may be related to the early stage of China's tourism industry development, and complete marketization may not be conducive to TFP. TI is significant at the 1% statistical level, and the coefficient is negative. This indicates that the transportation infrastructure level is not conducive to TFP of urban agglomerations.

## Conclusion

Based on the hypothesis and the cases of China's 10 urban agglomerations, this paper constructs a theoretical and empirical analysis framework for the impact of TIA on TFP. The analysis framework not only examines the mechanism and impact of TIA on TFP, but also examines the moderating effect of the public epidemic on this impact. In summary, this paper draws some main conclusion.

First, the agglomeration development of tourism industry in Chinese urban agglomerations is obvious, and the regional differences are gradually shrinking.

Second, a U-shaped relationship exists between TIA and TFP of Chinese urban agglomerations. The result indicates that the impact of TIA on TFP of Chinese urban agglomerations is first the congestion effect and then the agglomeration effect (a U-shaped curve). This shows that China's current tourism industry should release the development dividend so as to be the growth point of China's economic development.

Third, the public epidemic plays a moderating role in the relationship between TIA and TFP of urban agglomerations. The public epidemic has enhanced the congestion effect of TIA and weakened the agglomeration effect of TIA. This also shows that the public epidemic has a significant impact on the tourism industry and the TFP of urban agglomerations.

In order to continuously improve TFP of China's urban agglomeration, it requires various efforts. First, the urban agglomeration should promote the integration process of tourism industry development and strengthen the agglomeration effect of TIA on TFP. Urban agglomerations must remove barriers to cross-regional development, strengthening internal coordination, and enhance external collaboration to promote the effective flow of tourism resources. Second, urban agglomerations should vigorously promote the global tourism development model, which can not only realize the sharing of tourism resources dividends between regions, but also allow tourists to enjoy cross-provincial tourism experience services. Third, in the era of normalization of the epidemic, urban agglomerations should activate the form of online tourism and promote the development of smart tourism. The smart tourism system should be fully connected to the public health management system to provide “reassurance” for both tourists and tourism enterprises. It is necessary to realize the integration of online and offline as soon as possible, give full play to the function of technology to adjust cultural distance, and strongly support the integration of culture and tourism. Fourth, the technological innovation level of tourism industry and new tourism products should be continuously improved. Finally, we must give full play to the role of the government in macroeconomic regulation and improve the marketization institution. The marketization institution is a double-edged sword. It is necessary to foster strengths and avoid weaknesses so as to provide institutional guarantees for the development of urban agglomerations.

However, further research is needed as follows. First, the scientific calculation of the total factor productivity of urban agglomerations. If the indicators, data, and methods are different, the measurement results of the total factor productivity of the urban agglomeration will show large differences. In the follow-up study, it is necessary to select more realistic indicators, and use different methods to measure total factor productivity of urban agglomerations. The second is the study of the spatial effects of tourism industry agglomeration. In the future research, spatial location factors will be introduced to analyze the impact of tourism industry agglomeration on neighboring areas. Third, there are many factors affecting the development of tourism industry. In the future research, more factors will be considered.

## Data Availability Statement

The original contributions presented in the study are included in the article/supplementary material, further inquiries can be directed to the corresponding author.

## Author Contributions

The work done on the project was distributed among JW and JM. JW designed the analytical characterization, empirical study frame, and provided the critical revision and editing. JM analyzed the data and evaluated the results. Both authors contributed to writing the paper. Both authors contributed to the article and approved the submitted version.

## Funding

The authors acknowledge the financial supports from Soft Science Project of Suzhou Agriculture and Rural Bureau; Soft Science Project of Suzhou Agricultural Modernization Research Center.

## Conflict of Interest

The authors declare that the research was conducted in the absence of any commercial or financial relationships that could be construed as a potential conflict of interest.

## Publisher's Note

All claims expressed in this article are solely those of the authors and do not necessarily represent those of their affiliated organizations, or those of the publisher, the editors and the reviewers. Any product that may be evaluated in this article, or claim that may be made by its manufacturer, is not guaranteed or endorsed by the publisher.
